# COVID-washing of ultra-processed products: the content of digital marketing on Facebook during the COVID-19 pandemic in Uruguay

**DOI:** 10.1017/S1368980021000306

**Published:** 2021-01-26

**Authors:** Lucía Antúnez, Florencia Alcaire, Gerónimo Brunet, Isabel Bove, Gastón Ares

**Affiliations:** 1 Instituto Polo Tecnológico de Pando, Facultad de Química, Universidad de la República, ByPass de Rutas 8 y 101 s/n, Pando, Canelones, CP 91000, Uruguay; 2 Espacio Interdisciplinario, Universidad de la República, Montevideo, Uruguay; 3 UNICEF Uruguay, Montevideo, Uruguay

**Keywords:** Food marketing, Policymaking, Food environment, Regulation

## Abstract

**Objective:**

To explore the use of references to the COVID-19 pandemic as part of the marketing strategies used on Facebook to promote ultra-processed products.

**Design:**

A search for Facebook accounts of ultra-processed products was performed using a master list of products commercialised in two online supermarkets in Uruguay. For each of the identified Facebook accounts, all the content posted from the confirmation of the first cases of COVID-19 in Uruguay, on 14 March 2020, until 1 July 2020 was recorded. Posts including mentions to COVID-19, social distancing measures or their consequences were identified and analysed using content analysis.

**Setting:**

Uruguay, Latin America.

**Results:**

A total of 135 Facebook accounts were identified, which generated a total of 1749 posts related to ultra-processed products, from which 35 % included references to COVID-19. The majority of the posts included references to prevention measures. Approximately one-third of the posts included proposals of activities to do at home, most of which were linked to a healthy lifestyle. Tips for coping with quarantine and descriptions of the charitable work undertaken by brands were also identified.

**Conclusions:**

Results from the present work provide evidence that industries of ultra-processed products have taken advantage of the COVID-19 pandemic to promote their products, create positive associations with the brands and improve their image as part of their digital marketing strategies.

Social distancing measures aimed at minimising physical contact between individuals or population groups have been the core component of the public health response to curb the pandemic of COVID-19 worldwide^(1,2)^. These measures have disrupted daily life and have created a series of psychological and economic challenges^(3–5)^. In particular, COVID-19 has motivated several changes in eating habits^(6,7)^. Studies conducted in different countries worldwide have shown that one of such changes is an increase in the consumption of ultra-processed foods with excessive content of sugars, fat and sodium in a substantial proportion of the population^(6–9)^. Consumption of ultra-processed products has been associated with obesity and non-communicable diseases, which can increase the risk for severe complications of COVID-19^(10,11)^.

While multiple factors have contributed to increasing consumption of ultra-processed products during the COVID-19 pandemic, the influence of marketing cannot be ignored. Marketing can be regarded as a cue to consume that makes products more salient in consumers’ mind^(12)^. Scientific evidence has shown that marketing influences product recognition, food preferences, food choice and eating behaviour, particularly among children and adolescents^(12–14)^.

In the last decade, the contribution of digital marketing to the marketing mix of food companies has grown, partly motivated by the increased popularity of social media and content-sharing platforms^(15,16)^. This type of marketing is characterised by its ubiquity, uninterrupted availability and interactivity, which makes it more persuasive and impactful than traditional marketing^(17,18)^. Digital marketing has been particularly relevant during the COVID-19 pandemic, as people were more exposed to this type of marketing due to the increased use of social media during social distancing^(6,19)^. In this sense, previous studies have shown that ultra-processed products are frequently marketed in social media using marketing features that increase interaction and engagement, such as promotions, interactive games and user-generated content^(20,21)^.

The food industry has quickly responded to changes in consumer behaviour and has intensified online advertisement to promote their products and gain competitive advantages^(22,23)^. Marketing analysts have recommended brands to stay active in social media and to show themselves as emphatic and supportive, for example, by showing support to communities during the pandemic or supporting social distancing^(24)^. Such messages are expected to create positive associations and provide legitimacy, which increases consumers’ trust in the brand, quality perceptions, purchase intention and brand loyalty^(25,26)^.

This strategic marketing approach can be regarded as ‘COVID-washing’, in resemblance to greenwashing. Greenwashing can be defined as the dissemination of symbolic and misleading information by an organisation to show an environmentally responsible public image, without any substantive action^(25,27)^. In the context of ultra-processed products, COVID-washing can be regarded as the dissemination of symbolic information about the contributions of companies of such products to health and well-being. This strategy has the potential to ‘camouflage’ the negative health impact of ultra-processed products^(27)^.

In this context, the present work intends to contribute to an in-depth understanding of how the food industry used the COVID-19 pandemic as part of their digital marketing strategies to promote ultra-processed products. The study was conducted in Uruguay. As of October 2020, Uruguay was the only Latin American country who had managed to avoid an exponential increase in COVID-19 cases without mandatory quarantine^(28)^. The focus of the study was placed on Facebook, the most popular social media in the country^(29)^. In particular, the study aimed at answering the following research questions: (i) Were references to the COVID-19 pandemic included as part of the marketing strategies used on Facebook to promote ultra-processed products in Uruguay? and (ii) what type of messages were conveyed as part of the digital marketing strategies of ultra-processed products in Facebook?

Results are expected to provide relevant insights for policymakers given increasing concerns over digital marketing of unhealthy foods, particularly when targeted at children and adolescents^(16,30,31)^. Although some governments have implemented policies to protect children from the harmful effects of marketing of unhealthy foods, most of the policies are not comprehensive in nature and do not apply to social media or are not clearly designed^(32,33)^. In the specific case of Uruguay, although a law was passed in 2014 to ban marketing of foods with high content of sugar, fat and sodium targeted to children on broadcast media (television and radio)^(34)^, the regulatory decree needed for its effective implementation has not been issued yet.

## Methods

### Data collection

First, a comprehensive master list of ultra-processed products commercialised in the country was generated through a review of the two largest and most popular online supermarkets (Disco and Tienda Inglesa). Ultra-processed products were defined as ‘*formulations of ingredients, mostly of exclusive industrial use, typically created by series of industrial techniques and processes*’^(35)^. The following categories of ultra-processed products were considered: sweetened beverages; dairy products; bread, crackers and cookies; breakfast cereals and cereal bars; savoury snacks; sweets and chocolates; desserts and ice creams; cured meats; sweet spreads and jams; soups and bouillon cubes; frozen meals. The products included in each of the categories are shown in the online supplementary material, Supplemental Table S1. A master list including product name, brand and manufacturer was generated through a review of all the products commercialised in the two online supermarkets within the selected categories.

Using the master list, a search for Facebook accounts was performed using the search tool considering the brand and manufacturer names as keywords. Only Uruguayan accounts were considered. After this search, a total of 135 Facebook accounts were identified. For each Facebook account, all the content posted from the confirmation of the first cases of COVID-19 in Uruguay on 14 March 2020 until 1 July 2020 was recorded. Seven of the accounts corresponded to brands that commercialised ultra-processed products but were mainly oriented to the commercialisation of the other groups of the NOVA classification^(35)^. Given that the emphasis of the work was on the digital marketing strategies used to promote ultra-processed products, posts related to natural or minimally processed foods (meat, rice, frozen vegetables), culinary ingredients (spices) or processed foods (cheese, pasteurised tomato puree) were not considered (*n* 88).

### Data analysis

Posts including mentions to COVID-19, social distancing measures or their consequences were identified following a data triangulation approach. First, one of the researchers who authored the study classified the posts. Subsequently, the classification was revised by two additional researchers, and disagreements were resolved by open discussion with a third researcher.

All posts related to COVID-19 were analysed using content analysis^(36)^ following a data triangulation approach. One of the researchers involved in the study examined the posts and analysed their content using a combined deductive/inductive coding approach^(28)^. Using a deductive approach, the following themes were selected for the analysis: objective of the post, target audience, marketing techniques included in the post and references to COVID-19. Then, an inductive approach was used to identify categories within each of the themes. For this purpose, one of the researchers identified categories for each of the themes as they emerged when examining the visual and textual elements of the posts. The coding was revised by another researcher, who checked for inconsistencies. The procedure described above was used to resolve disagreements between the two researchers. Content analysis was performed manually. A detailed description of the themes and categories is included in the online supplementary material, Supplemental Table S2.

Descriptive statistics are reported in the manuscript. The relative frequency of occurrence of each category was calculated by dividing the number of posts related to that specific category by the total number of posts referencing COVID-19. Examples of posts were selected for each of the identified themes. All the analyses were performed in Spanish, and selected texts were translated for publication.

## Results

As shown in Table 1, ninety-six of the 135 Facebook accounts posted content in the period elapsed from the confirmation of the first cases of COVID-19 in Uruguay until 1 July 2020. Most of the accounts corresponded to four categories of ultra-processed products: sweetened beverages; sweets and chocolates; bread, crackers and cookies; and dairy products. The accounts generated a total of 1749 posts related to ultra-processed products, from which 35 % (*n* 619) included a reference to COVID-19, social distancing measures or their consequences.

**Table 1:** Number of Facebook accounts of ultra-processed products and posts generated in the period elapsed from the confirmation of the first cases of COVID-19 in Uruguay, on 14 March 2020, until 1 July 2020, for all the accounts and disaggregated by category

Category	Number of Facebook accounts (*n*)	Number of accounts who posted content (*n*)	Total number of posts (*n*)	Percentage of the posts including references to COVID-19 (%)^*^
Sweetened beverages	26	20	333	49
Sweets and chocolates	26	14	301	31
Bread, crackers and cookies	22	13	219	19
Dairy products	18	15	265	32
Frozen meals	11	9	143	49
Cured meats	8	6	167	35
Breakfast cereals and cereal bars	7	6	87	33
Savoury snacks	7	4	63	19
Desserts and ice cream	6	5	72	28
Boullion cubes and soups	3	3	56	50
Sweet spreads and jams	1	1	43	16
Total	135	96	1749	35

* The percentage of all the posts generated by the Facebook accounts in the period elapsed from the confirmation of the first cases of COVID-19 in Uruguay, on 14 March 2020, until 1 July 2020, that included references to COVID-19, prevention measures or their consequences.

### Objective of the posts

**Fig. 1.**
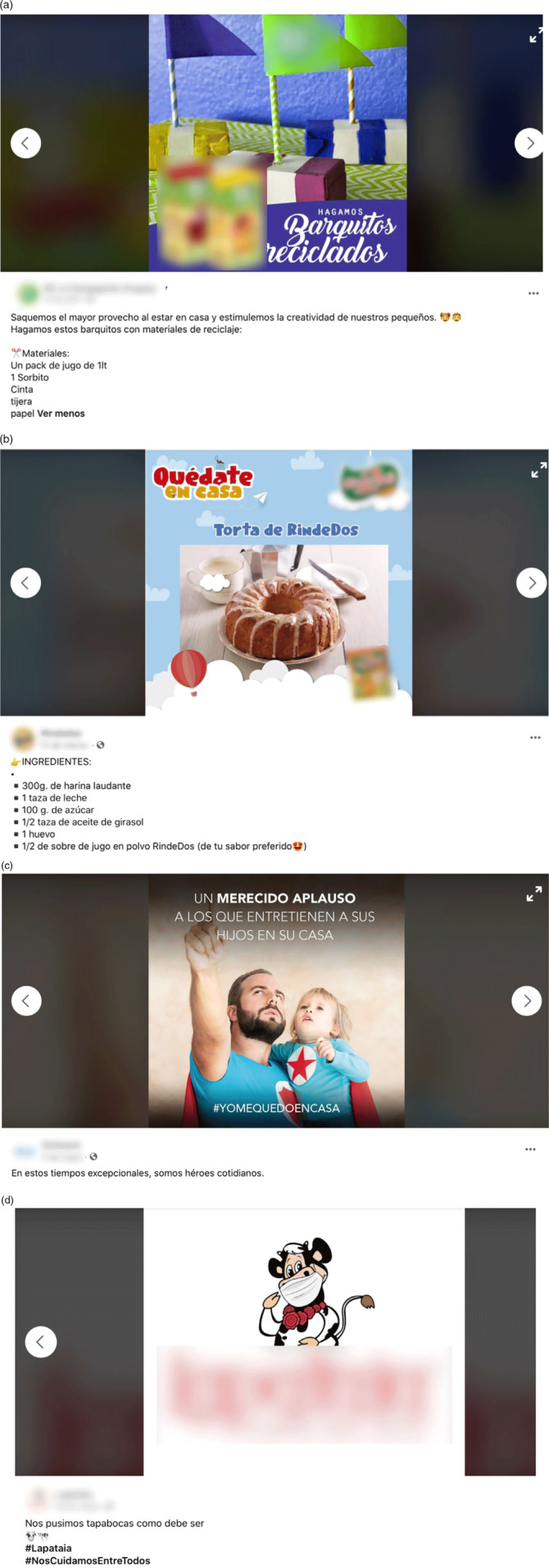
Example of Facebook posts (a, b) promoting consumption of specific ultra-processed products and (c, d) brands of ultra-processed products in the context of COVID-19. Note: The translation of the Spanish text of the posts correspond to: (a) Let’s make recycled boats. Let’s take advantage of staying at home and let’s stimulate the creativity of our little ones. Let’s make these boats with recycled materials: 1 juice package (1 L), 1 straw, tape, scissors, paper; (b) Stay at home. (Name of the brand) cake. Ingredients: 300 g self-rising flour, 1 cup of milk, 100 g of sugar, 1/2 cup of sunflower oil, 1 egg, 1/2 package of (Name of brand) powdered juice (your favourite flavour); (c) a deserved applause to those who entertain their children at home #stayathome. In these exceptional times, we are everyday heroes; (d) we are wearing facemask as it should be #(name of the brand) #wetakecareofeachother

Posts sought two main objectives: promoting consumption of specific products (54 % of the identified posts) or promoting the brand (46 % of the identified posts). As shown in Fig. 1, posts promoting consumption of specific products included visual and/or textual references to those products (Fig. 1a and b), whereas posts promoting the brands did not include any reference to specific products and only referred to the brand in the context of pandemic (Fig. 1c and d). No major differences were found between them in the themes analysed in the present work (target audience, marketing techniques, references to COVID-19). For this reason, in the following sections, results are presented at the aggregate level.

### Target audience

**Fig. 2.**
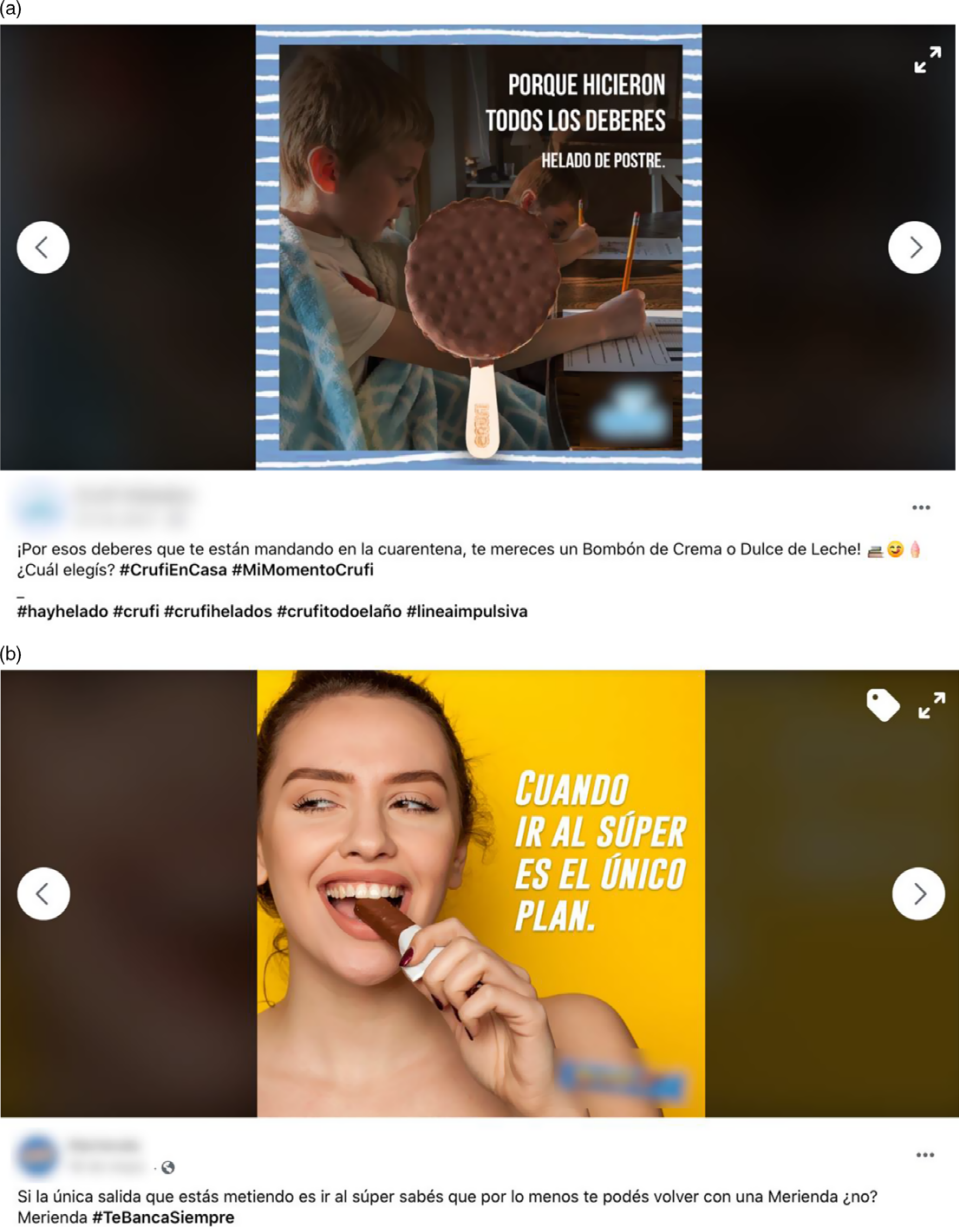
Example of Facebook posts promoting consumption of specific ultra-processed products targeted at (a) children and (b) adolescents and young adults. The translation of the Spanish text of the posts correspond to: (a) because they did all the homework. Ice cream as dessert. Because of all the homework they are sending you during the quarantine, you deserve a (name of the product). Which one do you choose? #(name of the brand)athome #MyMoment(name of the brand) #thereisicecream #(name of the brand) #(name of the brand)allyearlong #impulsive line; (b) when going to the supermarket is the only plan. If you are only going out to the supermarket you know that at least you can come back with a (name of the product), right? #StandsByYouAlways

The majority of the posts targeted the general audience (Table 2). However, 22 % of the posts targeted a specific group using pictures or textual references. Parents with children were the most frequently targeted group, followed by adolescents and young adults. Figure 2 shows two posts promoting the consumption of specific ultra-processed products targeted at these two groups. The post targeted at parents with children promoted the consumption of ice cream to reward children for doing their homework (Fig. 2a), whereas the post targeted at adolescents and young adults promoted the consumption of a sweet snack in the context of the lack of plans (Fig. 2b). Posts targeted at people who practice sports and women were also identified (Table 2)

**Table 2:** Content analysis of the posts that included references to COVID-19

Theme/category	Percentage of the posts^*^
Objective of the post	
Promoting consumption of specific products	54
Promoting the brand	46
Target audience	
General audience	78
Parents with children	10
Adolescents and young adults	7
People who practice sports	4
Women	1
Marketing techniques	
Pictures	62
Videos	38
Conversations with users	23
Competitions and prizes	15
Corporate social responsibility	12
Online shopping/delivery	7
Events	4
Special price promotions	2
References to COVID-19	
Prevention measures	65
Proposals of activities to do at home	36
References to taking care of each other	25
Tips for coping with quarantine	8
Encouraging messages	6
Description of charitable work	6
Commitment of the brand to accompany customers	6

* The table shows the percentage of the identified posts (*n* 619) coded in each of the categories for each of the selected themes. A detailed description of the themes and categories is included in the online supplementary material, Supplemental Table S2.

### Marketing techniques

A wide range of marketing techniques were identified (Table 2). All the posts included pictures or videos. Pictures were more frequently included in the posts that promoted consumption of specific ultra-processed products compared to the posts that promoted the brands, which more frequently included videos. Approximately one-fourth of the posts intended to establish conversations with the users, encouraging them to send comments about their proposals or suggestions on how to consume their products, to share the activities they were doing to cope with social distancing measures or to recommend movies, series or books.

Competitions were included in 15 % of the posts. The prizes associated with most of the competitions were sets of ultra-processed products from the brand, sometimes accompanied by other non-food products (e.g. tickets for drive-in cinema). The rest of the competitions included products to overcome quarantine as prize (e.g. tickets for a streaming show).

Social responsibility was used as a marketing technique in 12 % of the posts. The accounts highlighted different actions to convey the idea that the brands were actively trying to contribute to society. The posts referred to the commitment of the brand to assuring food availability (e.g. ‘*We keep giving our best so that you can enjoy our products from home*’ or ‘*Our commitment with taking quality foods to all Uruguayans keeps increasing*’). Donations of ultra-processed products to the most vulnerable populations through different non-governmental organisations and similar organisations were also mentioned.

Some of the accounts promoted delivery and online shopping as a contribution to stay-at-home recommendations (e.g. ‘*stay at home, we take our products to your house*’). Events were also identified as a marketing technique in the posts, as brands organised online concerts, virtual conversations or virtual workshops. Finally, special price promotions of specific ultra-processed products were highlighted by 2 % of the posts.

### References to COVID-19

As shown in Table 2, 65 % of the posts included references to COVID-19 through the inclusion of references to prevention measures as their central message (e.g. Fig. 1d), as well as a secondary message using a hashtag (e.g. #stayathome, #quedateencasa in Spanish). Stay-at-home recommendations and social distancing were the most frequently mentioned prevention measures, accounting for 55 % of all the posts related to this category. A minority of posts referred to wearing facemasks, avoiding sharing personal objects, washing hands, sneezing into the elbow, ventilation and no-touch greetings. In some of the posts, references to prevention measures were used to promote individual consumption of products through expressions such as ‘*not sharing is being responsible*’, ‘*you don’t give bad vibes, you are responsible (*when not sharing the product)’ and ‘*we meet and each one has its* (product name)’. In addition, 25 % of the posts stressed the need to take care of each other in the context of the pandemic.

Approximately one-third of the posts included proposals of activities to do at home, most of which were linked to a healthy lifestyle (e.g. cooking or exercise). Cooking at home was the most frequently mentioned activity in the posts targeted at promoting consumption of specific products. The posts included recipes or suggestions of simple culinary preparations involving ultra-processed products, such as bouillon cubes, cream cheese, cured meats or powdered drinks (e.g. Fig. 1b). The majority of the posts intended to create associations between ultra-processed products and home-made meals through expressions such as ‘*the true flavour is at home*’ or ‘*home-made food is made with love and dedication*’. In particular, one of the posts stated that ‘*children who cook develop healthier habits*’ while promoting consumption of a product with excessive content of sugar (chocolate-flavoured powder drink). Suggestions for at-home exercise routines were also identified. This was the case of all the posts from sport drink brands, which proposed exercise routines while promoting consumption of sugar-sweetened beverages as part of the routine. Suggestions of activities for children were also identified, including handcrafts and games involving empty packages (e.g. Fig. 1a) and science fairs. At-home activities also included invitations to events, such as online concerts, online stand-up shows, virtual conversations or virtual workshops.

Accounts provided tips for coping with quarantine in 8 % of the posts. The tips included following a healthy diet, doing workouts at home and spending time doing enjoyable activities. Although in a small number, some posts directly associated consumption of ultra-processed products to coping with quarantine (e.g. Fig. 2b). In addition, 6 % of the posts included encouraging messages to raise positive emotions and associations (e.g. *‘We will win this fight together’)* or to thank the medical community and volunteers involved in response to COVID-19 (e.g. *‘The world will be always thankful that you have not taken a break in this moment. Thank you. Dear volunteers from community diners for leaving everything to help those in need’).*

**Fig. 3.**
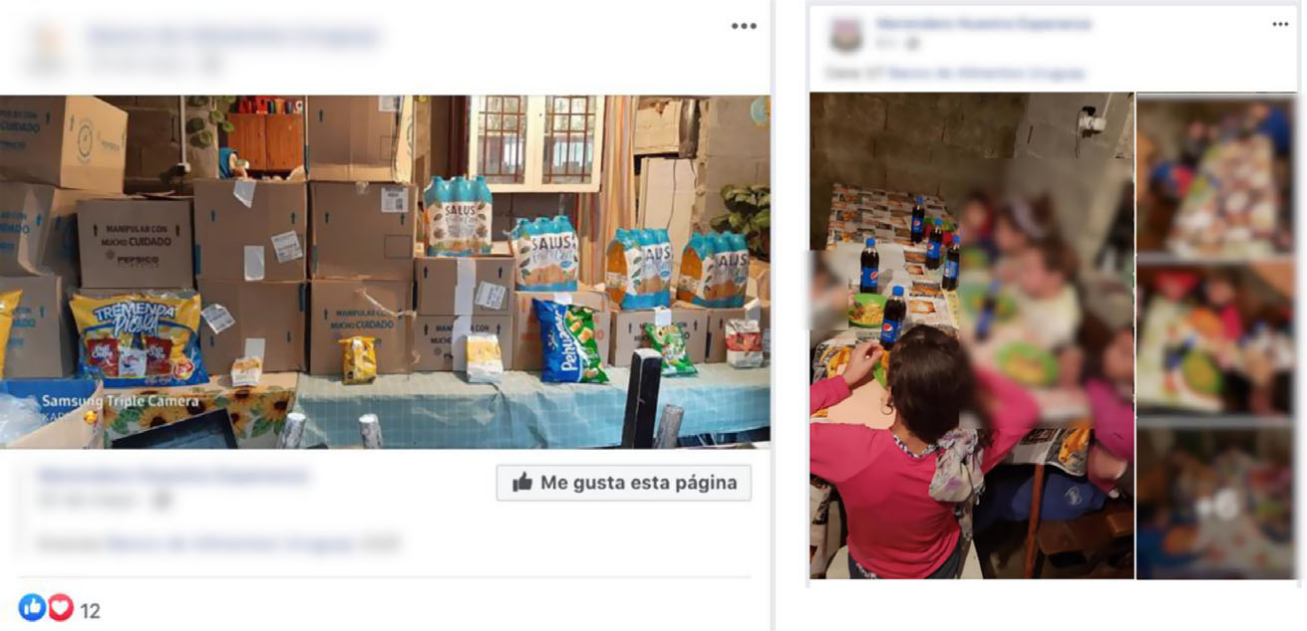
Example of Facebook posts from non-governmental organisations (NGO) showing donations from brands of ultra-processed products

As shown in Table 2, 6 % of the posts described charitable work undertaken by brands in the context of the economic crisis generated by COVID-19. The posts stressed that the brands donated products to those in need through non-governmental organisations. Using a snowball approach, the Facebook accounts of the non-governmental organisations were assessed. Although a detailed analysis was not performed, the posts allowed to identify that the donations mainly comprised ultra-processed products (e.g. sweetened beverages, snacks, sweets and chocolates, cookies, instant soups) that ended up being consumed by the most vulnerable segments of the childhood population (Fig. 3). Finally, the brands also stressed their commitment to accompanying customers in the context of COVID-19 (e.g. ‘*We always accompany you*’, ‘(Brand name) *is with you at home*’).

## Discussion

The COVID-19 pandemic has created health, economic and social challenges worldwide^(37)^. The industry of ultra-processed products has reacted to those challenges by implementing a series of actions to stay competitive and gain profit, including changes in their marketing, promotion and distribution strategies^(38)^. In this context, the present work analysed the marketing strategies used by the industry of ultra-processed products on Facebook during the first 4 months of the pandemic in Uruguay. Results from the present work showed that the industry quickly adapted their digital marketing strategies on Facebook to reference the health and social concerns raised by the COVID-19 pandemic in 35 % of their posts.

Content analysis of the posts evidenced that companies intended to appear active and contributing to society in the context of the pandemic, in line with recommendations from marketing analysts^(22,24)^. As expected, a wide range of marketing strategies were identified in the posts, which frequently relied on the interactive nature of social media. The content of the posts frequently used references to prevention measures and social distancing to promote the consumption of ultra-processed products, as previously described by other authors across the globe^(23,38)^. These marketing strategies could exacerbate the adverse impact of the negative emotional consequences of COVID-19 on eating habits, promoting the consumption of ultra-processed products^(6–10,39,40)^. It is worth highlighting that half of the posts intended to promote the brand, without making any specific reference to specific products. A branding approach to food marketing has been already reported for both traditional and digital advertising^(16,41)^. This approach can increase recall and recognition of the brands and create positive associations, which subsequently increases purchase intention of the products commercialised by the brands^(13,42)^.

A relevant proportion of the posts identified in the study mentioned activities related to a healthy lifestyle. This represents a clear opposition to the negative effects of the promoted products on health, well-being and the environment^(43,44)^. In this sense, some of the posts encouraged home-made cooking using ultra-processed products as key ingredients or consumption of such products after working out. These results suggest that the industry of ultra-processed products relied on ‘COVID-washing’, that is, used the adverse context generated by the COVID-19 pandemic to improve their image and to create positive associations. Based on research on greenwashing, it can be hypothesised that COVID-washing can create positive associations with the brands, which can lead to an increase in consumers’ trust in the brand and purchase intention of their products^(25,26)^. Further research is needed to generate empirical evidence of the impact of COVID-washing on consumers’ perception of the brand and their purchase intention of ultra-processed products.

The posts also evidenced the use of social responsibility as a marketing strategy, as brands intended to show interest in social objectives beyond profit^(45)^. Some of the companies described the charitable work they had performed in the context of the pandemic, mainly associated with donations of ultra-processed products through non-governmental organisations. Similar strategies have been reported in several countries worldwide^(23,38)^. Through donations, the industry of ultra-processed products managed to achieve several strategic objectives^(38)^. First, they effectively distributed products that were close to expiration in the context of reduced sales. Second, they promoted their products among the most vulnerable populations by providing them free of cost. Third, the promotion of actions linked to social responsibility could contribute to create positive associations with brands in the general public, which can increase brand loyalty and purchase intention^(26)^. Finally, donations provided the opportunity to the industry of ultra-processed products to shape the policy agenda by creating the impression that they can provide solutions to overcome the health challenges created by COVID-19^(38)^. In this sense, policymakers and civil society should be aware of the acute conflicts of interest that underlie the aforementioned actions due to the negative health effects of ultra-processed products. Similar concerns have been risen for corporate social responsibility actions in the alcohol and gambling industries^(45)^.

### Policy implications of the findings

To date, only few countries worldwide have managed to implement regulations on digital marketing^(32,33)^. Results from the present work suggest the need to refine most regulations in order to adequately protect consumers from the potentially deleterious effects of digital marketing of ultra-processed foods.

Traditionally, most voluntary and mandatory regulations have focused on marketing of products with high content of sugar, fat and sodium and have excluded restrictions of brand marketing^(30,32,33)^. Results from the present work showed that a branding approach to digital marketing was highly prevalent during the COVID-19 pandemic in Uruguay, suggesting that a relevant part of digital marketing efforts was devoted to create positive associations with the brands. This marketing approach is not harmless; it has been shown to influence consumers’ associations and purchase intention of the products commercialised by the brands^(13,42)^. Therefore, the findings reported in this study reinforce the idea that brand marketing should be a central part of digital marketing regulations. The regulation of brand marketing is expected to be challenging as it requires the definition of which brands are associated with unhealthy products. A first step in this direction is the UK’s restriction of brand marketing that intends to promote specific products high in fat, salt or sugar^(46,47)^. Nevertheless, it should be noted that most of the social media posts promoting brands would not be included in this regulation. Policymakers are encouraged to engage in the challenging task of including brand marketing in social media within the scope of advertising regulations.

A second limitation of the current regulations is their focus on marketing ‘targeted at’ or ‘appealing to’ children and adolescents^(30,32,33)^. Most of the posts identified in the present work were targeted at the general audience. This suggests that focus on children and adolescents may not be enough to protect them from exposure to digital marketing of ultra-processed products or their negative consequences. In order to capture all the digital marketing children may be exposed to, regulations on digital marketing of ultra-processed foods should also include marketing targeted at mixed audiences. This is particularly relevant considering that effects of digital marketing are expected to be exacerbated by word-of-mouth effects, as users can share the content generated by the brands^(15–18)^. Policymakers should be aware that regulation of digital marketing targeted at the wide audience is expected to face fierce opposition due to concerns over infringement of legal rights of companies and censorship of commercial expression^(48,49)^.

### Limitations of the study

The limitations of the present research should be acknowledged. The study involved the evaluation of Facebook accounts from a single country, selected by researchers based on the products available in a limited number of categories in two online supermarket. Further research should confirm and expand the findings of the present work to a larger number of accounts across countries and social media platforms (e.g. Instagram). In this sense, the comparison of the content of digital marketing according to the stringency of the social distancing measures implemented by countries is a relevant avenue for future research.

Another limitation of the study is that it only analysed the content posted by the brands on their Facebook accounts and did not consider advertisements presented to Facebook users while using the platform. However, it should be acknowledged that the analysis of Facebook advertisements is not an easy task given the characteristics of digital marketing, particularly in terms of audience targeting strategies.

Finally, data on engagement with the posts, for example, number of likes, shares and comments, were not collected. The analysis of this information could contribute to evaluate the level of interaction with the content posted on Facebook by the industry of ultra-processed foods during the COVID-19 pandemic. This is particularly relevant considering that social media posts have the potential to go viral and reach an exponential number of users.

## Conclusion

Results from the present work provide evidence that industries of ultra-processed products have taken advantage of the COVID-19 pandemic to promote their products, create positive associations with the brands and improve their image as part of their digital marketing strategies. This suggests the need to implement multi-faceted strategies to raise awareness of the marketing strategies of ultra-processed products among stakeholders to increase political will to dismantle corporate actions that may exacerbate the negative effects on the COVID-19 pandemic on health and well-being worldwide.

## Supplementary material

For supplementary material accompanying this paper visit https://doi.org/10.1017/S1368980021000306. click here to view supplementary material

## References

[1] Wilder-Smith A & Freedman DO (2020) Isolation, quarantine, social distancing and community containment: pivotal role for old-style public health measures in the novel coronavirus (2019-nCoV) outbreak. J Travel Med 27, taaa020. 32052841 10.1093/jtm/taaa020 PMC7107565

[2] European Centre for Disease Prevention and Control (2020) *Considerations Relating to Social Distancing Measures in Response to COVID-19 – Second Update.* European Centre for Disease Prevention and Control; available at https://www.ecdc.europa.eu/sites/default/files/documents/covid-19-social-distancing-measuresg-guide-second-update.pdf (accessed April 2020).

[3] Douglas M , Katikireddi SV , Taulbut M et al. (2020) Mitigating the wider health effects of covid-19 pandemic response. BMJ 369, m1557. 32341002 10.1136/bmj.m1557 PMC7184317

[4] Brooks SK , Webster RK , Smith LE et al. (2020) The psychological impact of quarantine and how to reduce it: rapid review of the evidence. Lancet 395, 912–920. 32112714 10.1016/S0140-6736(20)30460-8 PMC7158942

[5] Arora T & Grey I (2020) Health behaviour changes during COVID-19: a mini review. J Health Psychol 25, 1155–1163. 32551944 10.1177/1359105320937053

[6] Scarmozzino F & Visioli F (2020) Covid-19 and the subsequent lockdown modified dietary habits of almost half the population in an Italian sample. Foods 9, 675. 10.3390/foods9050675 PMC7278864 32466106

[7] Górnicka M , Drywién ME , Zielinska MA et al. (2020) Dietary and lifestyle changes during covid-19 and the subsequent lockdowns among polish adults: a cross-sectional online survey PLifeCOVID-19 study. Nutrients 12, 2324. 10.3390/nu12082324 PMC7468840 32756458

[8] Steele EM , Rauber F , Costa CS et al. (2020) Dietary changes in the NutriNet Brasil cohort during the covid-19 pandemic. Rev Saude Publica 54, 91. 32901755 10.11606/s1518-8787.2020054002950 PMC7454165

[9] Ammar A , Brach M , Trabelsi K et al. (2020) Effects of COVID-19 home confinement on eating behaviour and physical activity: results of the ECLB-COVID19 international online survey. Nutrients 12, 1583. 10.3390/nu12061583 PMC7352706 32481594

[10] Gao F , Zheng KI , Wang X-B et al. (2020) Obesity is a risk factor for greater COVID-19 severity. Diabetes Care. doi: 10.2337/dc20-0682. 32409499

[11] Garg S , Kim L , Whitaker M et al. (2020) Hospitalization rates and characteristics of patients hospitalized with laboratory-confirmed coronavirus disease 2019 — COVID-NET, 14 states, March 1–30, 2020. Morb Mortal Wkly Rep 69, 458–464. 10.15585/mmwr.mm6915e3 PMC7755063 32298251

[12] Boyland EJ , Nolan S , Kelly B et al. (2016) Advertising as a cue to consume: a systematic review and meta-analysis of the effects of acute exposure to unhealthy food and nonalcoholic beverage advertising on intake in children and adults. Am J Clin Nutr 103, 519–533. 26791177 10.3945/ajcn.115.120022

[13] Kelly B , King M , Psy L et al. (2015) A hierarchy of unhealthy food promotion effects: identifying methodological approaches and knowledge gaps. Am J Public Health 105, e86–e95. 25713968 10.2105/AJPH.2014.302476 PMC4358159

[14] Mills SDH , Tanner LM & Adams J (2012) Systematic literature review of the effects of food and drink advertising on food and drink-related behaviour, attitudes and beliefs in adult populations. Obes Rev 14, 303–314. 10.1111/obr.12012 23297736

[15] Montgomery KC , Chester J , Grier SA et al. (2012) The new threat of digital marketing. Pediatr Clin North Am 59, 659–675. 22643172 10.1016/j.pcl.2012.03.022

[16] Boyland E , Thivel D , Mazur A et al. (2020) Digital food marketing to young people: a substantial public health challenge. Ann Nutr Metab 76, 6–9. 10.1159/000506413 32101856

[17] Buchanan L , Kelly B , Yeatman H et al. (2018) The effects of digital marketing of unhealthy commodities on young people: a systematic review. Nutrients 10, 148. 10.3390/nu10020148 PMC5852724 29382140

[18] Kelly B , Vandevijvere S , Freeman B et al. (2015) New media but same old tricks: food marketing to children in the digital age. Curr Obes Rep 4, 37–45. 26627088 10.1007/s13679-014-0128-5

[19] Sheth J (2020) Impact of Covid-19 on consumer behavior: will the old habits return or die? J Bus Res 117, 280–283. 32536735 10.1016/j.jbusres.2020.05.059 PMC7269931

[20] Freeman B , Kelly B , Baur L et al. (2014) Digital junk: food and beverage marketing on Facebook. Am J Public Health 104, 56–64. 10.2105/AJPH.2014.302167 PMC4232106 25322294

[21] Horta PM , Rodrigues FT & dos Santos LC (2018) Ultra-processed food product brands on Facebook pages: highly accessed by Brazilians through their marketing techniques. Public Health Nutr 21, 1515–1519. 29444739 10.1017/S1368980018000083 PMC10261659

[22] Nielsen (2020) Recalibrated consumption dynamics in a COVID-19 altered world. https://www.nielsen.com/eu/en/insights/article/2020/recalibrated-consumption-dynamics-in-a-covid-19-altered-world/ (accessed December 2020).

[23] White M , Nieto C & Barquera S (2020) Good deeds and cheap marketing: the food industry in the time of COVID-19. Obesity 28, 1578–1579. 32441869 10.1002/oby.22910 PMC7280662

[24] Broad M (2020) I want to break free! How are COVID ads effectively resonating? https://www.nielsen.com/apac/en/insights/article/2020/i-want-to-break-free-how-are-covid-ads-effectively-resonating/ (accessed December 2020).

[25] Walker K & Wan F (2012) The harm of symbolic actions and green-washing: corporate actions and communications on environmental performance and their financial implications. J Bus Ethics 109, 227–242.

[26] Abid T , Abid-Dupont M-A & Moulins J-L (2019) What corporate social responsibility brings to brand management? The two pathways from social responsibility to brand commitment. Corp Soc Resp Env Ma 27, 952–936.

[27] Ramus CA & Montiel I (2005) When are corporate environ mental policies a form of green-washing? Bus Soc 44, 377–414.

[28] Taylor F (2020) Uruguay is winning against covid-19. This is how. BMJ 370, m3575. 32948599 10.1136/bmj.m3575

[29] Instituto Nacional de Estadística (2019) Encuesta de Usos de Tecnologías de la Información y la Comunicación 2019 [Survey on the Use of Information and Communication Technologies]. Montevideo: Instituto Nacional de Estadística.

[30] WHO Regional Office for Europe (2018) Tackling Food Marketing to Children in a Digital World: Trans-Disciplinary Perspectives. Copenhagen: WHO Regional Office for Europe.

[31] Clark H , Coll-Seck AM , Banerjee A et al. (2020) A future for the world’s children? A WHO–UNICEF–lancet commission. Lancet 395, 605–658. 32085821 10.1016/S0140-6736(19)32540-1

[32] Kraak VI , Vandevijvere S , Sacks G et al. (2016) Progress achieved in restricting the marketing of high-fat, sugary and salty food and beverage products to children. Bull World Health Organ 94, 540–548. 27429493 10.2471/BLT.15.158667 PMC4933136

[33] Smith Taillie L , Busey E , Mediano Stoltze F et al. (2019) Governmental policies to reduce unhealthy food marketing to children. Nutr Rev 77, 787–816. 31329232 10.1093/nutrit/nuz021 PMC7528677

[34] Poder Legislativo (2014) Ley N° 19307. Ley de medios. Regulación de la prestación de servicios de radio, televisión y otros servicios de comunicación audiovisual [Law 19307. Regulation of the Provision of Radio, Television and Other Audiovisual Communication Services]. Montevideo: IMPO.

[35] Monteiro C , Cannon, G , Lawrence M et al. (2019) Ultra-Processed Foods, Diet Quality, and Health Using the NOVA Classification System. Rome: FAO.

[36] Krippendorff K (2004) Content Analysis: An Introduction to Its Methodology, 2nd ed. Thousand Oaks, CA: Sage Publications.

[37] Hevia C & Neumey A (2020) A Conceptual Framework for Analyzing the Economic Impact of COVID-19 and Its Policy Implications. New York: United Nations.

[38] Collin J , Ralston R , Hill SE et al. (2020) Signalling Virtue, Promoting Harm: Unhealthy Commodity Industries and COVID-19. NCD Alliance, SPECTRUM.

[39] Sominsky L & Spencer SJ (2014) Eating behavior and stress: a pathway to obesity. Front Psychol 5, 434. 24860541 10.3389/fpsyg.2014.00434 PMC4026680

[40] Hill DC , Moss RH , Sykes-Muskett B et al. (2018) Stress and eating behaviors in children and adolescents: systematic review and meta-analysis. Appetite 123, 14–22. 29203444 10.1016/j.appet.2017.11.109

[41] Boyland EJ & Halford JCG (2013) Television advertising and branding. Effects on eating behaviour and food preferences in children. Appetite 62, 236–241. 22421053 10.1016/j.appet.2012.01.032

[42] Boyland EJ & Christiansen P (2015) Brands and food-related decision making in the laboratory: how does food branding affect acute consumer choice, preference, and intake behaviours? A systematic review of recent experimental findings. J Agric Food Ind Org 13, 45–54.

[43] Pagliai G , Dinu M , Madarena MP et al. (2020) Consumption of ultra-processed foods and health status: a systematic review and meta-analysis. Br J Nutr 125, 308–318. 32792031 10.1017/S0007114520002688 PMC7844609

[44] Swinburn BA , Kraak VI , Allender S et al. (2019) The global syndemic of obesity, undernutrition, and climate change: the lancet commission report. Lancet 393, 791–846. 30700377 10.1016/S0140-6736(18)32822-8

[45] Geiger B & Cuzzocrea V (2017) Corporate social responsibility and conflicts of interest in the alcohol and gambling industries: a post-political discourse? Br J Sociol 68, 254–272. 28369716 10.1111/1468-4446.12249

[46] Advertising Standards Authority (2014) Code of Non-Broadcast Advertising and Direct and Promotional Marketing (CAP Code). London: Advertising Standards Authority.

[47] Committee of Advertising Practice (2017) *Identifying Brand Advertisement That has the Effect of Promoting an HFSS Product.* Advertising Guidance; available at https://www.asa.org.uk/asset/6B42B9F3–96EC-4A66-A9B50F0E21D845BF/ (accessed December 2020).

[48] Henriques P , Dias PC & Burlandy L (2014) Regulation of food advertising in Brazil: convergence and conflicts of interest. Cad Saude Publica 30, 1219–1228. 25099045 10.1590/0102-311x00183912

[49] Savell E , Fooks G & Gilmore AB (2015) How does the alcohol industry attempt to influence marketing regulations? A systematic review. Addiction 111, 18–32. 26173765 10.1111/add.13048 PMC4681589

