# Supplementary material for: COVID-washing of ultra-processed products: the content of digital marketing on Facebook during the COVID-19 pandemic in Uruguay
Source: Public Health Nutr. 2021 Jan 26:1–11. doi: 10.1017/S1368980021000306 (PMC7900666; doi:10.1017/S1368980021000306)
Supplement: Supplementary file 1 [file S1368980021000306sup001.pdf]

Supplementary material

Supplemental Table S1. Ultra-processed products included in each of the categories for creating the master list of products commercialized in two online supermarkets prior to the search for Facebook accounts.

Category	Products
Sweetened beverages	Carbonated soft drinks, juices, flavored waters, energy drinks, sport drinks, vegetable beverages (e.g. soy-based beverages, rice-based beverages), powdered drinks
Sweets and chocolates	Candies, chewing gum, jelly sweets, chocolates, *alfajores*
Bread, crackers and cookies	Toast bread and other types of packaged bread, crackers, cookies
Dairy products	Yogurts, flavoured milk, processed cheese, milk desserts
Frozen meals	Hamburgers, Pizza, *Empanadas*, Pre-fried potatoes, Breaded soy cutlet, Pasta
Cured meats	Cured meats, frankfurters, sausages
Breakfast cereals and cereal bars	Breakfast cereal, granola, cereal bars
Savory snacks	Potato crisps, biscuit-like snacks, tortilla chips, shaped crisp snacks, pretzels
Desserts and ice-cream	Frozen desserts, cakes, ice-creams
Boullion cubes and soups	Bouillon cubes, canned soups, dehydrated soups
Sweet spreads and jams	*Dulce de leche*, jams, hazelnut spreads

Notes: Alfajores are traditional Uruguayan products (sweet cookie sandwich filled with a layer of *dulce de leche*, usually covered with chocolate or meringue), whereas *dulce de leche* is a traditional type of sweetened condensed milk.

Supplemental Table S2. Description of the themes and categories considered in the content analysis of the Facebook posts

Theme	Category
Objective of the post: Describes the main objective the post intended to achieve.	*Promoting consumption of specific products:* Textual or visual references to specific products
	Promoting the brand: Visual or textual references to the brand without mentioning any specific product
Target audience: Main audience the post intends to reach.	*General audience*: Posts without any reference to any specific target group.
	Parents with children: Textual or visual references to children
	Adolescents and young adults: Textual or visual references to adolescents or young adults
	People who practice sports: Textual or visual references to sports.
	Women: Textual or visual references to adult women.
Marketing techniques: Describes the techniques used in the posts to promote the brand or specific products	*Pictures:* Inclusion of any type of digital images as part of the post
	Videos: Inclusion of videos as part of the post (published directly on Facebook or accessed through YouTube)
	Conversations with users: The account administrator intends to establish a conversation with users through the posts by asking questions or encouraging them to share comments, ideas or experiences
	Competitions and prizes: Description of competitions organized by the brands in which the users could gain different prizes
	Corporate social responsibility: Description of actions undertaken by the brands in the context of COVID-19 to contribute to society by assuring food availability or donating products
	Online shopping/delivery: Delivery or online shopping were described as a means to purchase the products
	Events: Description of events organized by the brands in which users could participate (e.g. concerts, virtual workshops)
	Special price promotions: Descriptions of price discounts or promotions for purchasing the products
References to COVID-19: Describes how COVID-19 and its consequences are referenced in the post	*Prevention measures:* Textual or visual references to measures to prevent the transmission of coronavirus (COVID-19)
	Proposals of activities to do at home: Description of ideas of activities that could be done at home during social distancing
	References to taking care of each other: Inclusion of references to the importance of taking care of each other in the context of the health and economic crisis generated by COVID-19
	Tips for coping with quarantine: Provision of tips for coping with quarantine or social distancing
	Encouraging messages: Inclusion of encouraging messages to raise positive emotions and associations in the context of the crisis generated by COVID-19
	Description of charitable work: Description of charitable work undertaken by the brands in the context of economic crisis generated by COVID-19
	Commitment of the brand to accompany customers: Description of the commitment of the brands with their customers in the context of the crisis generated by COVID-19
